# Analysis of factors influencing influenza outbreaks in schools in Taicang City, China

**DOI:** 10.3389/fpubh.2024.1409004

**Published:** 2024-07-19

**Authors:** Yao Shi, Lei Xu, Hai Jiang, Yongbin Cai, Changjun Bao, Wendong Liu

**Affiliations:** ^1^Taicang City Centre for Disease Control and Prevention, Suzhou, Jiangsu, China; ^2^Jiangsu Field Epidemiology Training Program, Jiangsu Provincial Centre for Disease Control and Prevention, Nanjing, Jiangsu, China; ^3^Jiangsu Provincial Centre for Disease Control and Prevention, Jiangsu Institution of Public Health, Nanjing, Jiangsu, China

**Keywords:** influenza, disease cluster, influenza vaccine, health knowledge, school outbreak

## Abstract

**Objective:**

This study aims to analyze the awareness of influenza prevention and control and the behavioral attitudes toward the work among parents and staff in schools in Taicang City and the impact of the vaccination rate among students on influenza outbreaks in schools. The findings can provide references for the development of effective control strategies for the spread of influenza.

**Methods:**

An anonymous questionnaire survey was conducted on 10,962 students from 20 schools in Taicang City, with class as the unit of analysis. The survey investigated their awareness of influenza prevention and control, their attitudes, and the vaccination coverage.

**Results:**

From January to June 2023, a total of 388 influenza outbreaks were reported in schools in Taicang City, involving 77 schools. There were 3,475 confirmed cases, with an average infection rate of 18.53%. In schools where influenza outbreaks had occurred, the incidence rate of those who received influenza vaccine was significantly lower than those who did not, and the vaccine protection rate was 28.22%. The knowledge awareness rates of “the main transmission routes of influenza” and “influenza vaccination can prevent influenza” among parents of students were 95.49 and 93.16%, respectively. The differences between schools involved in the epidemic and non-epidemic were statistically significant (*p* < 0.05). The correct attitudes of parents toward “actively reporting relevant symptoms to teachers when their children show symptoms” and “avoiding classes with diseases when their children are suspected to be sick” are 98.80 and 96.26%, respectively. The differences between schools with and without epidemic are statistically significant (*p* < 0.05). The correct attitudes of the class teacher toward “correct management and control of students with flu like symptoms in the class” and “taking correct prevention and control measures in the event of a flu epidemic in the class” were 89.36 and 92.55%, respectively. The differences between epidemic related and non-epidemic related classes were statistically significant (*p* < 0.05).

**Conclusion:**

Enhance the knowledge level of influenza prevention and control among parents of students, Strengthening the training for class teachers in emergency response to infectious diseases and increasing vaccination coverage among students can effectively reduce the incidence of influenza and thereby the occurrence of cluster outbreaks in schools.

## 1 Introduction

Influenza is an acute respiratory infectious disease caused by influenza viruses. It is mainly transmitted through droplets (1) and direct contact between individuals (2–4). It poses a serious threat to public health as it is highly contagious and can spread fast. Influenza outbreaks tend to occur in densely populated areas such as schools (5) and childcare facilities (6). In China, over 90% of reported influenza outbreaks occur in schools and childcare facilities (7). According to data from the Australian National Statutory Disease Surveillance System, the highest proportion of reported cases of influenza B were among children between 2001 and 2014 (8). Scholars in the United States have also shown that the gathering and activity environment on campus may lead to the rapid spread of influenza after its introduction, resulting in outbreaks (9).

Taicang City is located in the southeast of Jiangsu Province, on the south bank of the Yangtze River Estuary, with a total area of 809.93 square kilometers. The permanent population is 843,600, and the urbanization rate is 71.03%. Taicang City has 126 schools of all levels and types, including 64 kindergartens, 39 primary schools, 16 ordinary junior high schools, four ordinary high schools, one special education school, one secondary vocational school, and one higher vocational and technical college. There are 118,400 students in the city.

From January to June 2023, Taicang City in China’s Jiangsu Province witnessed a total of 431 outbreaks of infectious diseases in schools, of which 388 were influenza outbreaks, accounting for 90% of the total. Influenza outbreaks occurring in schools have become a major burden and challenge for infectious disease prevention and control in Taicang. Research on the factors influencing influenza outbreaks occurring in schools is lacking. This survey comprehensively analyzes the occurrence of influenza outbreaks in different levels and types of schools in Taicang City from January to June 2023. It investigates the impact of prevention and control measures taken in schools on the occurrence and development of influenza outbreaks and analyzes factors influencing influenza outbreaks in schools in Taicang City and vaccine protection rates to provide a basis for scientific prevention and targeted interventions.

## 2 Objects and methods

### 2.1 Research objects

Stratified sampling was used to select 10 schools (including five primary schools and five kindergartens) in Taicang City that experienced influenza outbreaks from January to June 2023 as the research objects, and a proportional sample of schools (five primary schools and five kindergartens) that did not experience influenza outbreaks during the same period was selected as the control group. The number of students, class teachers and school doctors participating in the study was 10,962, 188, and 28, respectively. They accounted for 13.84, 11.92, and 25.23% of the corresponding total population, respectively.

#### 2.1.1 Selection of school doctors

For schools with 1–2 school doctors, all school doctors are required to fill in a questionnaire. In schools with more than two school doctors, two were randomly selected to fill in the questionnaire.

#### 2.1.2 Selection of class teachers

In the survey schools, all classes with outbreaks were investigated. One class with no epidemic was randomly selected from each grade as a control. In the control schools, one class was randomly selected for each grade. The head teachers of the above classes are required to fill in the questionnaire.

#### 2.1.3 Selection of parents

One parent from each student in the selected class will be included in the survey and fill out the questionnaire.

### 2.2 Case definition

Influenza like cases are defined as those with fever (body temperature ≥ 38°C) and either cough or sore throat. The fever mentioned here should be in the course of an acute febrile illness, and body temperatures measured by both medical institutions and patients can be used for temperature determination.

### 2.3 Criteria for epidemic reporting

(1) Three or more new influenza like cases are found in a class in a day; (2) five or more new influenza like cases are found in a class in 3 days.《Monitoring Plan for Clustered Epidemic of Common Infectious Diseases in Schools in Taicang City》.

### 2.4 Investigation contents and methods

1. The data on influenza cluster outbreaks in schools comes from active monitoring of influenza cluster outbreaks in the jurisdiction, registration of epidemic reports, on-site epidemiological investigations, and disposal records.
2. The basic information of the school, the implementation of influenza epidemic prevention and control measures, and the mastery of influenza prevention and control knowledge by school doctors, class teachers, and parents were all obtained through questionnaire surveys.

### 2.5 Vaccination history

It refers to whether influenza vaccines have been administered since September 2022 (before the occurrence of the current illness).

### 2.6 Statistical analysis

Descriptive epidemiological analysis of influenza outbreaks and influenza vaccination coverage was conducted using SPSS 27.0. Comparison of rates and risk estimation were performed using the chi-square test (*χ*^2^-test). A *p*-value less than 0.05 indicates statistical significance.

## 3 Results

### 3.1 Overall situation of influenza outbreaks

From January to June 2023, a total of 388 influenza outbreaks occurring in schools were reported in Taicang City, including 96 in kindergartens (24.74%), 240 in primary schools (61.86%), 11 in secondary schools (2.84%), and 41 in technical secondary schools (10.57%). The influenza outbreaks were mainly concentrated in primary schools and kindergartens. The outbreaks affected 77 schools and 437 classes, with an average infection rate of 18.53% in classes. Specifically, the rate was the highest in kindergartens, hitting 19.73% (Table 1).

**Table 1:** Average infection rates in influenza outbreaks occurring in different levels of schools.

School level	Number of influenza outbreaks	Number of classes affected	Total number of students	Number of cases	Infection rate (%)
Kindergarten	96	97	3,395	670	19.73
Primary school	240	287	12,915	2,467	19.10
Secondary school	11	12	600	95	15.83
Technical secondary school	41	41	1,845	243	13.17
Total	388	437	18,755	3,475	18.53

**Figure 1:**
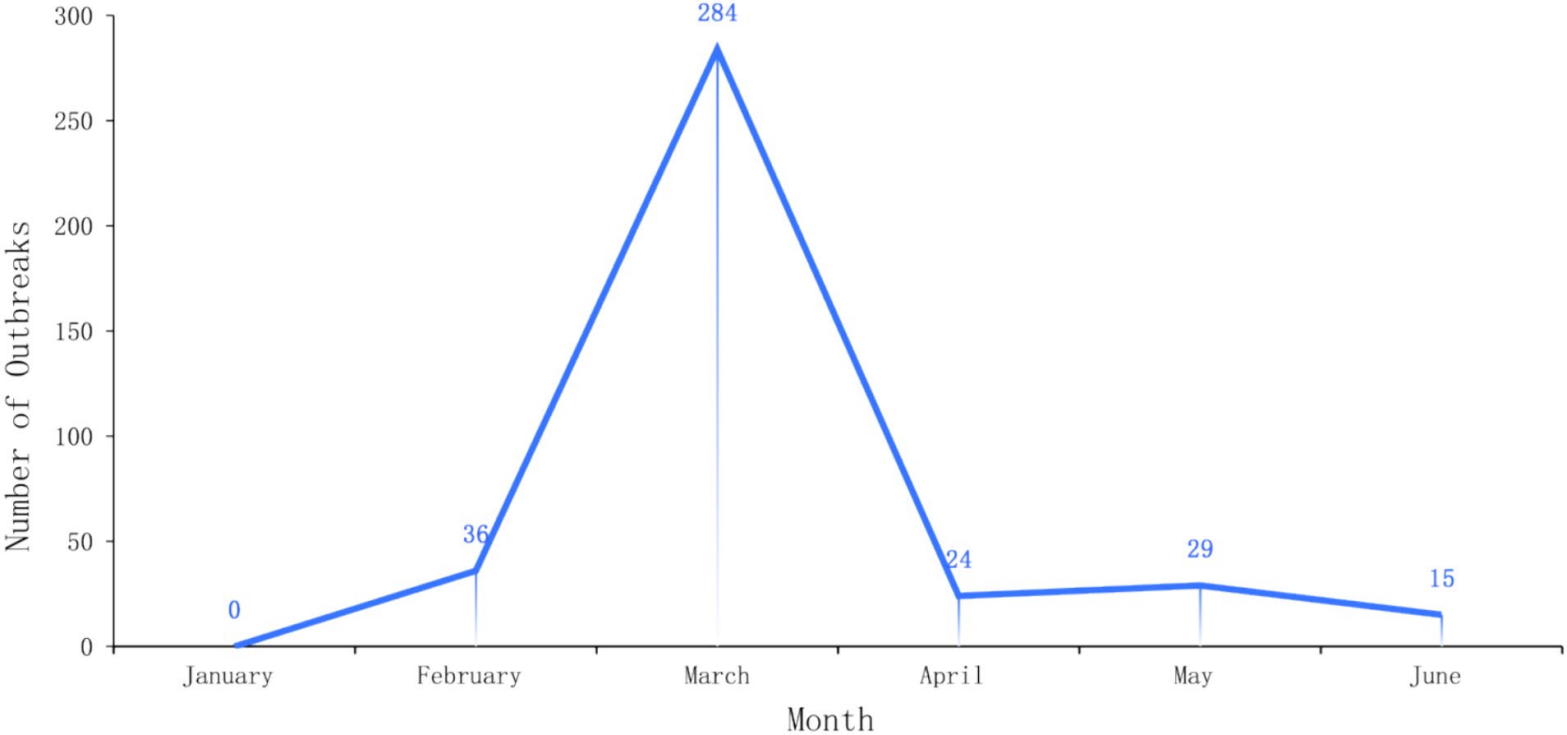
Time distribution of influenza outbreaks in different levels and types of schools in Taicang City from January to June 2023.

Each influenza cluster involved 3–29 cases, with 264 (68.04%) involving fewer than 10 cases, 110 (28.35%) involving 10–20 cases, and 14 (3.61%) involving more than 20 cases. Eight outbreaks affected three or more classes, 25 affected two classes, and the remaining 355 affected only one class. In the spring semester (from February to June), there was a peak in the number of influenza outbreaks in schools. A total of 284 cluster outbreaks occurred in March, accounting for 73.20% of the total number in the first half of the year. No influenza outbreaks were reported in January (Figure 1).

### 3.2 The impact of vaccinations on influenza outbreaks

Ten schools with a relatively large number of influenza cluster occurrences (≥5) were selected as the research objects from the 77 schools that experienced influenza outbreaks in Taicang City from January to June 2023. Ten schools were randomly selected as the control group from schools where no influenza outbreaks occurred during the same period.

The average vaccination rate in schools with influenza outbreaks was 15.14%, while that in schools without influenza outbreaks was 19.61%. The former was significantly lower than the latter (*χ*^2^ = 38.001, *p* < 0.05) (Table 2).

**Table 2:** Comparison of vaccination rates between schools where influenza outbreaks had or had not occurred.

Type of school	Number of students	Number of vaccinated students	Vaccination rate (%)
Schools with influenza outbreaks	5,454	826	15.14
Schools without influenza outbreaks	5,508	1,080	19.61
Total	10,962	1,906	17.39

At the class-level, the average vaccination rate in classes with influenza outbreaks was 13.16%, while that in classes without influenza outbreaks was 19.76%. The former was significantly lower than the latter (*χ*^2^ = 76.670, *p* < 0.05) (Table 3).

**Table 3:** Comparison of vaccination rates between classes where influenza outbreaks had or had not occurred.

Type of school	Number of students	Number of vaccinated students	Vaccination rate (%)
Classes where influenza outbreaks had occurred	3,944	519	13.16
Classes where influenza outbreaks had not occurred	7,018	1,387	19.76
Total	10,962	1906	17.39

Based on the analysis of classes from all selected relevant schools, the average vaccination rate was 20.33% in the surveyed schools without cluster outbreaks, while in the control schools without cluster outbreaks, the average vaccination rate was 19.61%. The difference is not statistically significant (*χ*^2^ = 0.391, *p* > 0.05) (Table 4).

**Table 4:** Comparison of vaccination rates between classes in schools without cluster outbreaks and control schools without cluster outbreaks.

Type of school	Number of students	Number of vaccinated students	Vaccination rate (%)
Investigating classes in schools that have not experienced influenza outbreaks	1,510	307	20.33
Compare classes in schools that have not experienced influenza outbreaks	5,508	1,080	19.61
Total	7,018	1,387	19.76

In the selected schools with influenza outbreaks, the average vaccination rate among influenza cases was 11.36%, while that among non-cases was 16.58%. The former was significantly lower than the latter (*χ*^2^ = 23.048, *p* < 0.05) (Table 5).

**Table 5:** Vaccination rates among cases and non-cases.

Type of school	Vaccination rate		*χ*^2^-value	*p-*value
	Cases (the rate)	Non-cases (the rate)		
Kindergarten	58(15.76%)	168(24.56%)	10.986	<0.05
Primary school	112(9.92%)	488(14.91%)	17.752	<0.05
Total	170(11.36%)	656(16.58%)	23.048	<0.05

### 3.3 Parents’ knowledge about influenza prevention and control and their behavioral attitude

#### 3.3.1 Parents’ knowledge of influenza prevention and control

Among the 10,962 parents surveyed, a total of 39,546 correct answers were provided for the four influenza-related knowledge questions, with an overall awareness rate of 90.19%. Among them, the awareness rate of “the main transmission routes of influenza” is the highest, reaching 95.49%, and the difference between the epidemic school group and the non-epidemic school group is statistically significant (*χ*^2^ = 29.734, *p* < 0.05). The awareness rate of “vaccination against influenza can prevent influenza” is 93.16%, and the difference between the epidemic school group and the non-epidemic school group is also statistically significant (*χ*^2^ = 15.989, *p* < 0.05). After dividing the surveyed subjects into epidemic related school group and non-epidemic related school group, it was found that a total of 5,454 parents of students in the epidemic related school group were surveyed, and 19,566 correct answers were given to 4 influenza related knowledge items, with a total awareness rate of 89.69%; A total of 5,508 parents of students in the non-epidemic school group were surveyed, and 19,980 correct answers were given to 4 influenza related knowledge items, with a knowledge rate of 90.69%; However, there was no statistically significant difference (*χ*^2^ = 1.108, *p* > 0.05) in the comparison of the awareness rates of “influenza epidemic season” and “which infectious disease influenza belongs to” between the epidemic school group and the non-epidemic school group in terms of related knowledge (Table 6).

**Table 6:** Comparison of the awareness rates of knowledge among parents.

Influenza-related knowledge	Group of people		Total	*χ*^2^-value	*p-*value
	Schools with influenza outbreaks	Schools without influenza outbreaks			
Influenza season	4,257(78.05)	4,253(77.21)	8,510(77.63)	1.108	0.293
Main transmission routes	5,149(94.41)	5,319(96.57)	10,468(95.49)	29.734	0.001
What kind of infectious disease is influenza?	5,132(94.10)	5,224(94.85)	10,356(94.47)	2.934	0.087
Vaccination can prevent influenza	5,028(92.19)	5,184(94.12)	10,212(93.16)	15.989	0.001

#### 3.3.2 Parents’ correct attitudes toward influenza prevention and control

Among the parents surveyed, totally 42,180 parents had a correct attitude toward influenza prevention and control, accounting for 96.20% of the total. Among them, the proportion of correct attitudes toward “children reporting relevant symptoms to teachers proactively” was the highest, reaching 98.80%, with a statistically significant difference (*χ*^2^ = 4.007, *p* < 0.05). The proportion of correct attitudes toward “children suspected to be ill and avoiding attending classes with illness” is 96.26%, with a statistically significant difference (*χ*^2^ = 19.631, *p* < 0.05). There was no statistically significant difference (*p* > 0.05) in the proportion of correct attitudes between the epidemic related school group and the non-epidemic related school group in terms of “avoiding contact with others during children’s quarantine period” and “avoiding taking children to crowded and polluted places during influenza epidemic period” (Table 7).

**Table 7:** Comparison of parents’ correct attitudes toward influenza prevention and control.

Relevant behavioral attitude	Group of people		Total	*χ*^2^-value	*p-*value
	Schools with influenza outbreaks	Schools without influenza outbreaks			
Report your child’s symptoms to the teacher	5,424(98.92)	5,460(99.12)	10,830(98.80)	4.007	0.045
Avoid contact with others while your child is in quarantine	5,184(95.05)	5,224(94.85)	10,408(94.95)	0.242	0.623
Avoid taking your child to class if there is a suspicion of illness	5,206(95.45)	5,346(97.06)	10,552(96.26)	19.631	0.001
Avoid taking your child to crowded places with dirty air during an influenza epidemic.	5,166(94.72)	5,224(94.85)	10,390(94.78)	0.086	0.770

### 3.4 Teachers’ knowledge of influenza prevention and control and their behavioral attitude

#### 3.4.1 Class teachers’ knowledge of influenza prevention and control

Among the class teachers surveyed, a total of 560 correct answers were provided for the four influenza-related questions, with an overall awareness rate of 74.47%. The awareness rate of influenza prevention methods is the highest (88.83%). The teachers from schools with influenza outbreaks gave 275 correct answers, with an overall awareness rate of 73.92%. Those from schools without influenza outbreaks gave 285 correct answers, with an awareness rate of 75.00%. The differences between the awareness rates of the two groups were not statistically significant (*χ*^2^ = 0.028, *p* > 0.05) (Table 8).

**Table 8:** Comparison of the awareness rates of knowledge among class teachers.

Influenza-related knowledge	Group of people		Total	*χ*^2^-value	*p-*value
	Classe teachers from schools with influenza outbreaks	Classe teachers from shools without influenza outbreaks			
The incubation period of influenza	72(77.42)	74(77.89)	146(77.66)	0.006	0.938
How influenza is spread?	81(87.10)	84(88.42)	165(87.77)	0.077	0.782
What kind of infectious disease is influenza?	40(43.01)	42(44.21)	82(43.62)	0.028	0.868
How to prevent influenza?	82(88.17)	85(89.47)	167(88.83)	0.080	0.777

#### 3.4.2 Class teachers’ correct attitudes toward influenza prevention and control

Among the class teachers surveyed, 702 instances held correct attitudes toward influenza prevention and control, accounting for 93.35% of the total. The proportion of correct control measures for students with flu like symptoms in the class is 89.36%, and the difference between epidemic related and non-epidemic related classes is statistically significant (*χ*^2^ = 8.346, *p* < 0.05). The proportion of “taking correct prevention and control measures when a flu epidemic occurs in the class” is 92.55%, and the difference is also statistically significant (*χ*^2^ = 5.125, *p* < 0.05). In terms of other attitudes, the proportion of asymptomatic infected individuals who need to adopt home isolation is the highest, accounting for 97.34%. The proportion of strict requirements for sick students to implement home isolation was 94.15%, however, there was no statistically significant difference in the holding of correct attitudes mentioned above (*χ*^2^ = 2.418, *p* > 0.05) (Table 9).

**Table 9:** Class teachers’ correct attitudes toward influenza prevention and control.

Relevant behavioral attitude	Group of people		Total	*χ*^2^-value	*p-*value
	Classe teachers from schools with influenza outbreaks	Classe teachers from schools without influenza outbreaks			
Correctly control the class when students present influenza-like symptoms	77(82.80)	91(95.79)	168(89.36)	8.346	0.004
Strictly require sick students to implement home quarantine	88(94.62)	89(93.68)	177(94.15)	0.075	0.784
Advocate for management and control measures for asymptomatic carriers	91(97.85)	92(96.84)	183(97.34)	0.184	0.668
Take correct prevention and control measures when an influenza outbreak occurs in the class	82(88.17)	92(96.84)	174(92.55)	5.125	0.024

### 3.5 School doctors’ knowledge of influenza prevention and control and their behavioral attitude

#### 3.5.1 School doctors’ knowledge of influenza prevention and control

Among the school teachers surveyed, a total of 81 correct answers were provided for the four influenza-related knowledge questions, with an overall awareness rate of 72.32% The question regarding the incubation period of influenza saw the highest awareness rate (92.86%). School teachers from schools where influenza outbreaks had occurred gave 36 correct answers, with an overall awareness rate of 64.29%. Those from schools where influenza outbreaks had not occurred gave 45 correct answers, with an awareness rate of 80.36%. The differences between the awareness rates of the two groups were not statistically significant (*χ*^2^ = 1.909, *p* > 0.05) (Table 10).

**Table 10:** School doctors’ knowledge of influenza prevention and control.

Influenza-related knowledge	Group of people		Total	*χ*^2^-value	*p-*value
	From schools with influenza outbreaks	From schools without influenza outbreaks			
The incubation period of influenza	12(85.71)	14(100.0)	26(92.86)	0.538	0.142
Main infection sources of influenza	4(28.57)	8(57.14)	12(42.86)	2.333	0.127
How influenza is spread?	11(78.57)	10(71.43)	21(75.00)	0.000	1.000
The best time to get vaccinated against influenza	9(64.29)	13(92.86)	22(78.57)	1.909	0.167

#### 3.5.2 School doctors’ correct attitudes toward influenza prevention and control

A total of 95 instances were recorded where the school doctors surveyed held correct attitudes toward influenza prevention and control, accounting for 84.82% of the total. Specifically, the rate of correctly managing and controlling students who had presented influenza-like symptoms was the highest, reaching 100% in both groups of schools. The proportion of school doctors advocating for home quarantine for asymptomatic carriers was the lowest, standing at 64.29%. The proportion of those who timely reported the situation when the criteria for school closure were met reached 82.14%, and those who performed influenza virus sampling at the best time accounted for 91.30%. The differences between the aforementioned corrected attitudes were not statistically significant (*χ*^2^ = 0.974, *p* > 0.05) (Table 11).

**Table 11:** School doctors’ correct attitudes toward influenza prevention and control.

Relevant behavioral attitude	Group of people		Total	*χ*^2^-value	*p-*value
	From schools with influenza outbreaks	From schools without influenza outbreaks			
Timely report the situation when the criteria for school closure are met	10(71.43)	13(92.86)	23(82.14)	0.974	0.324
Strictly control infected students	14(100.0)	14(100.0)	28(100.0)	–	–
Advocate for management and control measures for asymptomatic carriers	10(71.43)	8(57.14)	18(64.29)	0.622	0.430
Perform influenza virus sampling at the best time	13(92.86)	13(92.86)	26(92.86)	-	-

### 3.6 Implementation of influenza prevention and control measures in schools

A comparison was made regarding the implementation of 11 specific measures for infectious disease prevention and control. The results showed no significant difference in the implementation of prevention and control measures between the two groups of schools (Table 12).

**Table 12:** Main risk factors in schools with influenza outbreaks.

Prevention and control measures	Schools where influenza outbreaks had occurred		Schools where influenza outbreaks had not occurred		*χ*^2^-value	*p-*value
	Number	Rate (%)	Number	Rate (%)		
Student health check						
Checking of vaccination cards for daycare/entrance to school	10	100%	10	100%	–	–
Strict implementation of morning and afternoon checkups	10	100%	10	100%	–	–
Proper implementation of health check programs	9	90%	8	80%	–	–
Daily disinfection of public places						
Daily ventilation and disinfection of classrooms	8	80%	9	90%	–	–
Daily ventilation and disinfection of cafeterias	10	100%	10	100%	–	–
Case detection and control						
Timely isolation and control of sick students	7	70%	9	90%	–	–
Proper treatment for sick students	10	100%	10	100%	–	–
Proper treatment for sick teachers	9	90%	10	100%	–	–
Outbreak reporting and control						
Correct reporting of disease outbreaks	7	70%	9	90%	–	–
Correctly control of the cluster	10	100%	10	100%	–	–
Checking of the certificate of resumption of schooling after recovery	9	90%	10	100%	–	–

### 3.7 Reasons for students’ reluctance to receive the influenza vaccine

Among the 10,962 surveyed individuals, 1,906 (17.4%) had been vaccinated against influenza. Among the 9,056 individuals who had not received the vaccine, the reasons for their reluctance to be vaccinated, ranked from high to low, were as follows: “concern about vaccine safety” (4,226 individuals, 46.67%); “missed the centralized vaccination period” (2,136 individuals, 23.59%); “no unified organization by the school” (1,028 individuals, 11.35%); “child has contraindications for vaccination” (630 individuals, 6.96%); “physical discomfort during the scheduled vaccination period, not meeting the vaccination criteria” (322 individuals, 3.56%); “belief that the vaccine is not effective” (238 individuals, 2.63%); “already scheduled for vaccination, but the time has not arrived” (124 individuals, 1.37%); “unclear about how to schedule a vaccination” (118 individuals, 1.30%); “good physical condition, no need for vaccination” (90 individuals, 0.99%); “no time to take the child for vaccination” (64 individuals, 0.71%); “child is afraid of injections and unwilling to be vaccinated” (38 individuals, 0.42%); and “recently received another vaccine, and the doctor advised to wait for 6 months before receiving the influenza vaccine” (22 individuals, 0.24%); “flu vaccine need to pay for themselves” (20 individuals, 0.22%) (Table 13).

**Table 13:** Reasons for students’ reluctance to receive the influenza vaccine.

Reasons for students’ reluctance to receive the influenza vaccine	Number of people	Percentage (%)
Concern about vaccine safety	4,226	46.67
Missed the centralized vaccination period	2,136	23.59
No unified organization by the school	1,028	11.35
Child has contraindications for vaccination	630	6.96
Physical discomfort during the scheduled vaccination period, not meeting the vaccination criteria	322	3.56
Belief that the vaccine is not effective	238	2.63
Already scheduled for vaccination, but the time has not arrived	124	1.37
Unclear about how to schedule a vaccination	118	1.30
Good physical condition, no need for vaccination	90	0.99
No time to take the child for vaccination	64	0.71
Child is afraid of injections and unwilling to be vaccinated	38	0.42
Recently received another vaccine, and the doctor advised to wait for 6 months before receiving the influenza vaccine	22	0.24
Flu vaccine need to pay for themselves	20	0.22

## 4 Discussion

According to our study, influenza outbreaks in schools in Taicang City were concentrated in kindergartens and primary schools, accounting for 86.6% of the total number. In particular, cases in these two types of schools accounted for 90.3% of the total cases. This is consistent with reports from other regions (10–12), indicating that students in these schools may be more susceptible to influenza transmission (13), possibly due to their relatively weaker immune systems or more frequent contact with each other. In addition, the data showed that most outbreaks in schools were relatively small in scale, with 91.5% of the influenza outbreaks affecting only one class. This may be related to the decision to suspend classes when the local epidemic was found (14, 15). In terms of time distribution, there was a peak in March, with a total of 284 influenza outbreaks occurring, accounting for 73.20% of the total in the first half in 2023. This may be related to the seasonal nature of influenza transmission.

This study also shows that schools with influenza outbreaks had lower influenza vaccination rates in the study period, particularly in schools with more severe outbreaks where the average vaccination rate was only 15.14%. In comparison, schools without influenza outbreaks had a much higher average vaccination rate of 19.61%, suggesting that the vaccination may play a good role in preventing the spread of influenza in schools (16–18). Furthermore, in all schools with influenza outbreaks, the average influenza vaccination rate among cases was 11.36%, significantly lower than that among non-cases (16.58%). The statistically significant difference suggests the importance of influenza vaccination in reducing the occurrence and containing the spread of influenza. Vaccination, currently is the most economical and effective means of preventing influenza and its complications (19). Increasing influenza vaccination coverage in schools (20), especially in primary schools and kindergartens (21) is an important measure to effectively reduce the occurrence of influenza outbreaks in schools (22–24).

An analysis of the knowledge of influenza prevention and control showed that parents, class teachers, and school doctors demonstrated good overall awareness rates, with rates of 90.19, 74.47, and 72.32%, respectively. The awareness rates of “the main transmission routes of influenza” and “influenza vaccination can prevent influenza” among parents of students were 95.49 and 93.16%, respectively. There was a statistically significant difference (*p* < 0.05) between schools involved in the epidemic and non-epidemic, while no significant difference was found in the awareness rates of the four influenza related knowledge between the homeroom teacher and the school doctor. This indicates that the level of influenza knowledge among parents of students is an important factor leading to the occurrence of the epidemic. Therefore, in the future, it is necessary to pay more attention to improving the awareness level of influenza knowledge among parents of students, carrying out influenza knowledge promotion activities, and improving the overall level of disease prevention and control knowledge among the public. This will help to improve the overall effectiveness of influenza prevention and control in schools (25).

An analysis of correct attitudes toward influenza prevention and control showed that 96.20% of parents, 93.35% of class teachers, and 84.82% of school doctors held correct attitudes. In terms of school medicine, there is no significant difference between schools involved in the epidemic and non-epidemic schools. However, there is a statistically significant difference (*p* < 0.05) in the correct attitudes of parents toward “actively reporting relevant symptoms to the teacher when the child appears” and “avoiding classes with suspected illness when the child appears” between schools affected by the epidemic and non-epidemic. Similarly, there is a statistically significant difference (*p* < 0.05) in the correct attitude of the class teacher toward “correct control of students with flu like symptoms in the class” and “taking correct prevention and control measures in the class when a flu epidemic occurs” between epidemic and non-epidemic classes. This indicates that whether parents and homeroom teachers hold the correct attitude toward influenza prevention and control is an important factor leading to the outbreak of the epidemic. This indicates that in terms of epidemic prevention and control, it is necessary to strengthen the awareness of parents of students to actively report their children’s discomfort symptoms to teachers, avoid attending classes with illnesses, and reduce the spread and spread within the class. At the same time, further strengthen the emergency response training for class teachers, improve their ability to respond to the epidemic, and thus reduce the occurrence of class gatherings of epidemics (26). Studies have revealed that during influenza outbreaks, it is crucial to intensify education on personal hygiene for students, teachers, and parents, encourage them to develop good hygiene habits (27), and enhance the awareness of self-protection and prevention (28). Therefore, it is important to strengthen training in epidemic prevention and control and correct misconceptions, which contributes greatly to the effective management of infectious diseases in schools.

A comprehensive analysis of awareness and behavioral attitudes of parents, teachers, and school doctors on influenza prevention and control is conducted. There are two main reasons why influenza knowledge assessment scores of teachers and school doctors are lower than those of parents. Firstly, the focus of influenza prevention and control knowledge varies among parents, teachers, and school doctors. The difficulty of influenza knowledge tests on school doctors and teachers is relatively higher than that on parents, which may lead to parents having a better understanding of the illusion of influenza than teachers or doctors. Secondly, the survey results show that some school doctors do not have a medical background, and some are part-time school doctors. We have provided feedback on this issue to educational institutions and proposed improvement suggestions. Besides, we have jointly organized multiple lectures and training sessions on campus epidemic prevention and control knowledge with educational institutions, aiming to improve the professional level of school doctors. Uncovering problems is precisely the significance of our investigation. By identifying the problems, we can propose more measures of improvement.

An analysis of the implementation of influenza prevention and control measures in schools showed that although the results indicated no significant differences between the two groups of schools, this might be influenced by the small sample size or subjective response tendency of the respondents. However, other studies have shown that targeted risk management can effectively improve the implementation rate of prevention and control measures for infectious disease outbreaks in schools, lower the risk level, and thereby reduce the occurrence of disease outbreaks (29). The implementation of risk management for prevention and control of disease outbreaks in schools requires the schools to play a major role and take on the responsibility of improving organizational structures and hygiene facilities and implementing daily prevention and control measures (30). Strict implementation of morning and afternoon checkups, strict prevention of students attending classes or school when ill, effective management of potential sources of infection (31), and ventilation, disinfection, and health education measures are all key aspects of prevention and control (32). It is worth noting that schools must stay highly vigilant at any signs of disease outbreaks and measures such as early detection, reporting, isolation, and treatment should be strictly implemented, following the “early, careful, strict, and practical” prevention and control strategy (33). Early implementation of control measures (34) is crucial for reducing the incidence of influenza outbreaks and the infection rate in schools and is a key step in continuously reducing the risk of disease outbreaks (35, 36).

The analysis results of students’ willingness to receive the influenza vaccine indicate that the main reasons for their reluctance to be vaccinated include concerns about vaccine safety (46.67%), missed centralized vaccination periods (23.59%), the school not organizing unified vaccinations (11.35%), and children having contraindications for vaccination (6.96%). These factors reflect issues such as insufficient trust in the vaccine, inappropriate organization, and inappropriate scheduling during the vaccination process. To improve the influenza vaccination rate in schools, the following measures are recommended: Firstly, strengthen education and awareness on vaccine safety to address parents’ concerns and apprehensions. Secondly, arrange vaccination times reasonably to ensure that more students can receive the vaccine in a timely manner. Thirdly, recommend organizing unified vaccinations through schools to simplify the vaccination process and enhance convenience. Fourthly, personalized prevention and control advice should be provided for children with contraindications to ensure that they can also receive effective protection. Addressing these issues is crucial for comprehensively improving the effectiveness of influenza prevention and control. This will not only help increase the influenza vaccination rate in schools but also effectively reduce the spread of influenza and protect the health of students.

The analysis of various influencing factors reveals that the prevention and control of influenza outbreaks in schools is a complex and systematic undertaking. It requires not only the effective implementation by schools but also the collaboration of different stakeholders, such as the government, disease prevention and control agencies, and public health departments (37). Increasing vaccination coverage (38, 39), spreading influenza-related knowledge, and enhancing the emergency response capabilities (40) of class teachers are all effective intervention measures (41). In addition, scientific and precise risk management and timely adjustments to prevention and control measures are crucial for preventing and mitigating the impact of influenza outbreaks in schools. Furthermore, it is important to pay more attention to health education for teachers and parents of students and intensify the training of school doctors and health care teachers, so as to increase influenza vaccination rates in more schools (42, 43). Prevention and control strategies can be further optimized and innovated, and more specific and effective measures can be developed based on the actual situation of different schools to enhance the anti-epidemic level.

In conclusion, this study analyzed multiple aspects of influenza outbreaks occurring in schools in Taicang City, providing theoretical and practical references for future epidemic prevention and control. However, prevention and control work is an evolving process, and future research on disease outbreaks in schools will need to involve large samples to investigate the potential factors influencing influenza outbreaks in schools. It is also important to timely optimize strategies to be well-prepared for epidemic prevention and control efforts (44–46).

## 5 Limitations of this survey

1. Due to limitations in manpower and resources, the questionnaire items may not be precise enough. Some unclear or ambiguous questions might have affected the answers.
2. As the sample came from specific regions, schools, or grades, it cannot represent the nationwide level of influenza-related knowledge and awareness, leading to geographical bias.
3. The timing of the survey may have been influenced by seasonal influenza, as people’s attention to influenza and knowledge of it may vary across different seasons, affecting the generalizability of the results.
4. The answers from the respondents may be influenced by memory bias or subjective evaluation, leading to biased answers and potentially causing an overly optimistic or pessimistic estimation of influenza-related knowledge and awareness.
5. Since the survey relied mainly on questionnaires, it might not be possible to explore the respondents’ deeper understanding of influenza-related knowledge and comprehensively assess their understanding of and support for prevention and control measures.

## Data availability statement

The original contributions presented in the study are included in the article/Supplementary material, further inquiries can be directed to the corresponding authors.

## Ethics statement

This effort of disease control was part of CDC’s routine responsibility in Taicang City, China. Therefore, institutional review and informed consent were not required for this study. All data analyzed were anonymized.

## Author contributions

YS: Writing – original draft, Writing – review & editing, Formal analysis, Methodology. LX: Writing – original draft, Writing – review & editing, Investigation, Visualization, Data curation, Formal analysis. HJ: Writing – review & editing. YC: Writing – review & editing. CB: Writing – review & editing, Formal analysis, Methodology. WL: Writing – review & editing, Formal analysis, Methodology.
