# Supplementary material for: Analysis of factors influencing influenza outbreaks in schools in Taicang City, China
Source: Front Public Health. 2024 Jul 19;12:1409004. doi: 10.3389/fpubh.2024.1409004 (PMC11294167; doi:10.3389/fpubh.2024.1409004)
Supplement: Supplementary file 1 [file Data_Sheet_1.PDF]

Appendix 1

Survey of Influenza Epidemic Prevention and Control Measures and Implementation in Schools of Taicang City

(Filled in by school leaders)

No. □□□□□□□□

1. Basic Information of the school

(1)School Name

(2)Street/town to which it belongs

(3)School address

(4)School level:☐Kindergarten ☐Elementary ☐Middle School ☐High school

☐Secondary school ☐Junior College and above

(5)Nature of school:☐Public ☐Private

(6)School size: ___ classes; ___ staff members;___ students

(7)Health personnel allocation:___ school doctor (___full-time, ____part-time)

2.Students' Health Examination

(1) Does the school strictly adhere to the inspection of kindergarten/school vaccination certificates?

A. Yes

B. No (reason for not being implemented)

(2)"Under normal circumstances (in the absence of a cluster outbreak), how often should students undergo health checks (either in the morning or afternoon)?

A. Twice daily

B. Once per day

C. Once every three days

D. Not fixed

E. Other (please specify)"

(3）(Multiple choices) What is the procedure for conducting daily health checks?

A. Observing the student's mental state

B. Inquiring about the student's health

C. Recording absences due to illness

D. Other (please specify)

3. Daily disinfection of public places

(1) What is the frequency of classroom ventilation disinfection on a daily basis (when no cluster epidemic occurs)?

A. Once a day

B. Twice every 2 days

C. Three times every 3 days

D. Seven times every week

E. Other (please specify)

(2) What is the frequency of ventilation and disinfection in the canteen on a daily basis (in the absence of a cluster outbreak)?

A. Once a day

B. Twice every 2 days

C. Three times a week

D. Daily

E.Other (please specify)

4. Case discovery and control

(1)What is the expected timeframe for implementing control measures once a student is identified as having suspected influenza infection?

A. Immediately

B. Within 30 minutes

C. Within an hour

D. Within 3 hours

E. Within 6 hours

F. Other (please specify)

(2)How to manage students exhibiting flu-like symptoms?

A. Assess the student's wishes and make a decision regarding quarantine.

B. Advise the student to take protective measures and allow them to continue attending classes in school.

C. Notify the parents to pick up their child, seek medical attention promptly, and implement home isolation measures.

D. Other (please specify)

(3)How is staff management handled when flu-like symptoms occur?

A. The school has no explicit provisions and makes decisions regarding whether to continue teaching normally at its discretion.

B. Students are required to quarantine at home according to the standards of class suspension and may resume teaching after recovery.

C. Staff members are instructed to take protective measures (such as wearing masks) and continue teaching as usual.

D. Other (please specify)

5. Epidemic Reporting and Control

(1)(Multiple choices) What criteria will the school use to report an influenza outbreak to the community hospital/CDC?

A. 3 or more new influenza-like cases in the same class/dormitory in one day

B. 5 or more new influenza-like cases in the same class/dormitory within 3 days

C. 4 or more new influenza-like cases in the same class/dormitory within 3 days

D. 5 or more new influenza-like cases within one week in the same class/dormitory

E. Others (please specify)

(2) What control measures are generally in place when there is a cluster of influenza in a school?

A. Disinfect classrooms, dormitories, toilets and other areas where students have been present.

B. Strengthen morning check-ups to detect potential patients.

C. Open windows frequently to ensure proper ventilation in classrooms, dormitories, and canteens.

D. Suspend indoor crowd gathering activities at the school.

E. Strictly implement isolation treatment for sick students and prohibit their return to school before recovery.

F. Others (please specify)

(3) What is the process for students who have been suspended due to the flu before resuming classes?

A. Resume classes with a hospital recovery certificate.

B. Resume classes after meeting conditions set by the school doctor's examination.

C. Provide a hospital rehabilitation certificate for review by the school doctor before resuming classes.

D. Resume classes after meeting requirements checked by the homeroom teacher

E.Classes will be automatically resumed after the expiration of the isolation period

F.Others (please specify)
