# Supplementary material for: Analysis of factors influencing influenza outbreaks in schools in Taicang City, China
Source: Front Public Health. 2024 Jul 19;12:1409004. doi: 10.3389/fpubh.2024.1409004 (PMC11294167; doi:10.3389/fpubh.2024.1409004)
Supplement: Supplementary file 2 [file Data_Sheet_2.PDF]

Appendix 2

Survey on the Influenza Prevention and Control Capabilities of School Doctors in Taicang City

(Completed by School Doctors)

No. □□□□□□□□

1. Influenza Prevention and Control Knowledge

(1) The incubation period of influenza is defined as the time from human contact with pathogens to the appearance of obvious symptoms.

A. It is generally 1-7 days, with most being 2-4 days.

B. Generally 1-5 days, most 1-3 days.

C. Generally 1-14 days, most 7-10 days.

(2) (Multiple choice) The main source of infection for seasonal influenza is:

A. People with the flu

B. People with invisible infections

C. Carriers of the virus

D. Poultry

(3) Influenza viruses are mainly transmitted through respiratory secretions, but also through direct or indirect contact with mucous membranes such as the mouth, nose and eyes.

A. Airborne transmission

B. Contact transmission

C. Droplet transmission

D.Fecal-oral transmission

(4) The best time to get a flu shot is:

A.From January to March each year

B.From October to January each year

C.From June to September every year

2.Influenza Prevention and Control Behavior

(1) (Multiple choices) What criteria will the school use to report an influenza outbreak to the community hospital/CDC?

A. 3 or more new influenza-like cases in the same class/dormitory in one day

B. 5 or more new influenza-like cases in the same class/dormitory within 3 days

C. 4 or more new influenza-like cases in the same class/dormitory within 3 days

D. 5 or more new influenza-like cases within one week in the same class/dormitory

(2) Students who are sick at school while waiting to be sent home or to the hospital should

A. Wait in their original classroom and ask them not to go out

B. Move around freely on campus without restrictions

C. They should be transferred to the school isolation room for isolation

(3) What's wrong about the flu?

A. Influenza is A respiratory infection caused by the influenza virus

B. It mainly starts with fever, headache, sore throat and general discomfort. The body temperature can reach 39-40℃

C. Asymptomatic infected persons do not transmit influenza viruses

D.Children who are infected with influenza take a long time to detoxify and often pass the influenza virus to their classmates and their family members

(4) Influenza virus detection sampling, what is best collection time?

A.The day of onset

B.One week after onset of illness

C.The first three days, not more than seven days, after onset of illness

D.One week before onset of illness
