# Supplementary material for: Analysis of factors influencing influenza outbreaks in schools in Taicang City, China
Source: Front Public Health. 2024 Jul 19;12:1409004. doi: 10.3389/fpubh.2024.1409004 (PMC11294167; doi:10.3389/fpubh.2024.1409004)
Supplement: Supplementary file 3 [file Data_Sheet_3.PDF]

Appendix 3

Influenza Prevention and Control Knowledge Survey of School Teachers in Taicang City

(Completed by Classroom Teachers)

No. □□□□□□□□

1、Expertise in the prevention and control of influenza.

(1) The incubation period of influenza is defined as the time from human contact with pathogens to the appearance of obvious symptoms.

A. Generally 1-7 days, most 2-4 days

B. Generally 1-5 days, most 1-3 days

C. Generally 1-14 days, most 7-10 days

(2) Influenza virus is mainly transmitted through respiratory secretions, but also through oral, nasal, eyes and other mucous membranes direct or indirect contact.

A. Airborne transmission

B. Contact transmission

C. Droplet transmission

D. Fecal-oral transmission

(3)Influenza, abbreviated as flu, is a type of "()" infectious disease and is an acute respiratory infection caused by the influenza virus.

A. Class A

B. Class B

C. Class C

D. Other legally managed and key monitored infectious diseases

(4) The following statements about influenza prevention are incorrect:

A. Early use of antibiotics can be effective in preventing influenza

B. Good personal hygiene can also prevent influenza

C.Influenza vaccination is the most effective way to prevent influenza

D.Drug prophylaxis is not a substitute for vaccination

2.Influenza Prevention and Control Behavior

(1) When a student in the class exhibits symptoms such as fever, cough, runny nose, or sore throat, the appropriate course of action is to:

A. Immediately transfer the student to the school isolation room and report them to the school doctor for treatment according to professional guidance.

B. Observe the severity of symptoms and continue with class if they are mild.

C. In order not to disrupt the student's studies, they can wear a mask and continue attending class.

(2) If a student in the class is diagnosed with influenza, he/she must be strictly quarantined at home for 4 days. However, if the parents of the student are unable to care for him/her due to work commitments and wish for him/her to return to school, the appropriate course of action is ()

A. The student should be allowed to go home for a symbolic two-day rest and then return to school.

B. We understand the challenges that parents face, but considering the importance and urgency of epidemic prevention and control in schools, it is necessary for parents to independently find ways to overcome difficulties and ensure that students adhere to a

4-day home isolation period.

C. In order to minimize disruption to studies, the student will be seated in a designated area of the classroom and required to wear a mask at all times

(3) What is wrong with the following statement about influenza?

A. Influenza is a respiratory infection caused by the influenza virus.

B. It typically presents with symptoms such as fever, headache, sore throat, and general discomfort. Body temperature can reach 39~40℃.

C. Asymptomatic individuals infected with influenza can still transmit the virus.

D. Children infected with influenza may have a prolonged period of viral shedding and are at risk of transmitting the virus to their classmates and family members.

(4)(Multiple choices) When there is a cluster of influenza outbreaks in the class, which measures do you think should be taken?

A. Disinfect classrooms, dormitories, toilets and other areas where students have been present.

B. Enhance morning class inspections to identify potential patients.

C. Regularly open windows to ensure proper ventilation in classrooms, dormitories and canteens.

D. Suspend indoor crowd gathering activities at the school.

E. Enforce strict isolation treatment for sick students and prohibit their return to school until they have fully recovered.
