# Supplementary material for: Analysis of factors influencing influenza outbreaks in schools in Taicang City, China
Source: Front Public Health. 2024 Jul 19;12:1409004. doi: 10.3389/fpubh.2024.1409004 (PMC11294167; doi:10.3389/fpubh.2024.1409004)
Supplement: Supplementary file 4 [file Data_Sheet_4.PDF]

Appendix 4

Survey on Parents' Knowledge of Influenza Prevention and Control in Taicang City

(To be completed by parents of students)

No. □□□□□□□□

1.Child infection

(1) Has your child experienced flu-like symptoms (body temperature ⩾38℃, accompanied by cough or sore throat) from January 2023 to the present?

A. Yes

B. No

If yes, please indicate the date of onset of influenza: _____ year ___ month

(2)Prior to the onset of flu-like symptoms in your child, did any household members experience similar symptoms?

A. Yes

B. No

C. Unclear

(3)Prior to the onset of flu-like symptoms in your child, did any of their classmates exhibit similar symptoms?

A. Yes

B. No

C. Unclear

2.Knowledge of Influenza Prevention and Control

(1) The influenza epidemic season primarily occurs during the winter and spring, with no significant outbreaks in other seasons.

A. Correct

B. Incorrect

C. Unclear

(2) The influenza virus is primarily transmitted through respiratory droplets, which are produced when an infected person sneezes or coughs.

A. Correct

B. Incorrect

C. Unclear

(3) Influenza is an acute respiratory infection caused by the influenza virus.

A. Correct

B. Incorrect

C. Unclear

(4) Getting a flu shot is an effective measure for preventing the flu.

A. Correct

B. Incorrect

C. Unclear

3.Attitudes and Behaviors for Influenza Prevention

(1)During the school term, it is important for children to proactively inform their teacher if they experience any discomforting symptoms such as fever, sore throat, or fatigue.

A. Correct

B. Incorrect

C. Unclear

(2)When a child is ill and confined to home due to quarantine, they are unable to

attend classes. However, they are permitted to engage in outdoor play with neighbors and classmates.

A. Correct

B. Incorrect

C. Unclear

(3)Even if a child is diagnosed with the flu, and it is not a serious case, should the child still be kept in class?

A. Yes

B. No

C. Unclear

(4)Whenever possible, it is advisable to refrain from bringing children to crowded and unsanitary locations during a flu epidemic.

A. Correct

B. Incorrect

C. Unclear

4.Vaccination Status of Children

(1)Has your child received a flu vaccine from September 2022 to the present?

A. Yes, if so, please provide the date of vaccination (___Year____ month)

B. No

(2)If not vaccinated, select the reason for not being vaccinated? ()

A. Worry about adverse reactions and don't want to get vaccinated

B. The school is not uniformly organized

C. Missed centralized inoculation without reseeding

D. The child has contraindications

E. Others (please specify) ________
